# Arbuscular mycorrhizal fungi – a natural tool to impart abiotic stress tolerance in plants

**DOI:** 10.1080/15592324.2025.2525843

**Published:** 2025-07-09

**Authors:** Ishita Samanta, Kaustav Ghosh, Ruchita Saikia, Savita, Pooja Jha Maity, Gopal Chowdhary

**Affiliations:** a Plant Molecular Biology Laboratory, School of Biotechnology, Kalinga Institute of Industrial Technology (KIIT) Deemed to be University, Bhubaneswar, Odisha, India; b Department of Botany, Hansraj College, University of Delhi, Delhi, India

**Keywords:** Arbuscular mycorrhizal fungi, abiotic stress tolerance, signal transduction, strigolactones, symbiosis

## Abstract

Arbuscular mycorrhizal fungi (AMF) are crucial components of the soil microbiomes that establish symbiotic associations with most terrestrial plants. The review summarizes the basic mechanisms behind the plant-AMF symbiosis, the genes involved in the fungal and their plant counterparts, novel biomolecules and growth regulators, leading to probable signal transduction pathways. It also focuses on the involvement of lipids and strigolactones in establishing AMF-plant symbiosis. Herein, we further emphasize the role played by these AMF in enhancing plant resistance to various abiotic stresses while giving a broad outline of current research practices and attempting to dissect the mechanism behind the AMF-mediated abiotic stress signal transduction. Discussion on the mechanisms behind this stress reduction involving AMF will be valuable for the researchers, agronomists, and environmentalists involved in sustainable agriculture. Water scarcity, salinity, heavy metals, and extreme temperatures are the primary abiotic stresses that pose serious challenges to agricultural sustainability and ecosystem functioning. Conventional responses to such pressures typically rely on genetic modifications as well as chemical treatments, which could be expensive and detrimental to the environment. However, these AM fungi act in an alternative way that is natural and cost-effective too, leading to healthy plants with resilience toward stress through symbiosis, leading to the fulfillment of the United Nations Sustainable Development Goal (UNSDG) 2 of zero hunger.

## 1. Introduction

A mutually beneficial symbiotic association between fungi and plant roots is called mycorrhiza, where fungi colonize the plant roots and receive sugar and other organic compounds from the plant. Mycorrhizal associations are widespread among plants. Globally, approximately 83% of dicotyledonous and monocotyledonous plants form mycorrhizal relationships, while almost all gymnosperms have been observed to have mycorrhizal associations.^1^ Non-mycorrhizal associations are found in specific habitats or conditions such as very dry or saline soils, waterlogged areas, soils with extremely high or low fertility and severely disturbed habitats.^2^ It has been proposed that mycorrhizal associations are almost necessary for most terrestrial plants to have adequate growth and development.^1,2^ There are certain plant families where mycorrhizal associations are absent, such as Proteaceae, Cruciferae and Chenopodiaceae.^1^ Mycorrhizal associations could be two types – i. certain fungi making arbuscular/tree-like structures penetrating till the inner cortex of roots involved in the symbiotic nutrient exchange are referred to as arbuscular mycorrhiza (AM), ii. class of fungi present on the root’s external surface, where the fungal hyphae surround the root tips and are housed between the epidermal cells, referred to as ectomycorrhiza (EM).^3^ While most plants are thought to generate only one kind of mycorrhizal connection, some are able to create both types of associations within the same root system.^4^ Out of the two, AMF has drawn more attention lately and has been proven to be beneficial in multiple facets of plant life. Uptake of water and nutrients such as nitrogen, along with phosphorus in particular, by the host plant is enhanced by AMF; in exchange, the host plant supplies up to 20% of the carbon fixed through photosynthesis to the fungi.^5^

AMF are found in diverse ecosystems and extreme environments, based on which they can be classified as xerophilic, thermophilic, psychrophilic, acidophilic, alkalophilic, and halophilic.^6^ The most vital region of mycorrhizal interaction is the rhizosphere zone, which refers to a confined, small root-influenced area of the soil.^7^ The fungi, more specifically mycorrhizal fungi, form a symbiosis with the plant’s roots, thereby usually deriving sugar and other carbohydrates from the plants to use as a source of energy and in return they provide the plant with important micro and macro-nutrients.^8^

The roots of plants release hormones such as strigolactones, which are known for establishing symbiotic associations, particularly with AMF. Strigolactone acts as a hormone that attracts hyphae toward the root cells and stimulates their branching and metabolism.^9,10^ Plant hormones, particularly auxins (Indole-3-acetic acid, IAA), are crucial for the process of arbuscular growth in plants. Signaling molecules known as “Myc factors” are predominantly released by AMF in response to plant hormones, and they help to facilitate the establishment of the mycorrhizal association.^11,12^ This symbiotic relationship demonstrates a complex interaction between fungi and plants, where both partners benefit, enhancing plant nutrient acquisition, influencing plant signaling and even interacting with other organisms present in the ecosystem.

Increased soil carbon sequestration and reduction of soil erosion are some of the other important dimensions that AMF contributes to maintaining a productive and healthy environment for plant growth.^13^ Associated fungi provide several benefits to the plant and recent studies demonstrated their involvement in enhancing the plant’s ability to tolerate environmental stress. ^14–16^ Temperature plays a critical role in mycorrhizal association.^17^ Mycorrhiza helper bacteria support mycorrhizal symbiosis by promoting fungal colonization and nutrient uptake. However, deleterious bacteria can negatively impact mycorrhizal associations, highlighting the complexity of soil microbial communities.^18^ These interactions can enhance nutrient cycling, resistance to disease, and overall plant health. This review aims to address the role of AMF and its potential application to abiotic stress tolerance by plants.

**Figure 1.**
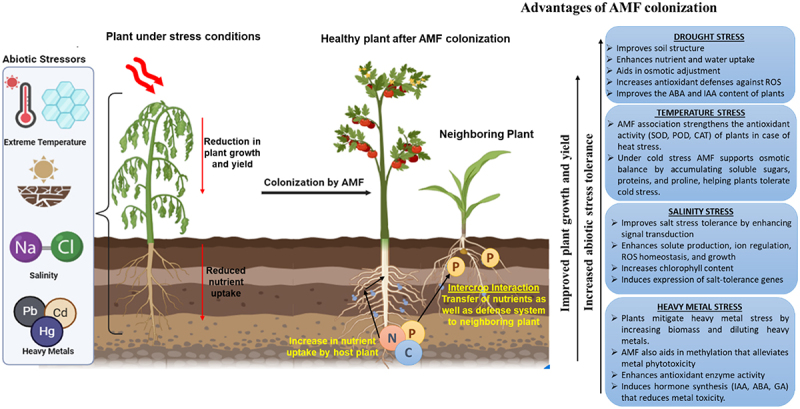
Overall attributes of AMF in protecting plants against abiotic stress conditions.

According to statistics, a symbiotic relationship with AMF is mainly formed with terrestrial plants, enhancing growth and tolerance to stress.^19^ AMF helps improve plant’s confrontation to abiotic stress factors like salinity, drought, temperature, and toxic heavy metals by absorption of nutrients and water, better root architecture, osmolyte buildup, and increasing stress-related gene expression (Figure 1).^20^ An increase in cellular reactive oxygen species (ROS) concentration is a major indication of stress conditions in plants. Polyamines are small aliphatic amines that impart abiotic stress tolerance by ROS scavenging and stabilizing the cell membranes.^21^ AMF controls the biosynthesis and regulation of polyamines (spermidine, spermine, and putrescine), thereby contributing to better stress tolerance. The activity and expression of aquaporins are also regulated by AMF through control over the passage of water across cellular membranes. This regulation ensures plant cells with efficient water uptake and transport, protecting plants from dehydration during drought and saline stress.^22,23^ AMF also modulates and activates phytohormone synthesis and antioxidant defense systems, offering a promising sustainable agriculture approach by potentially improving crop productivity and environmental safety to counter climate change and agricultural malpractices.^24,25^

## 2. The symbiotic relationship with AMF

The symbiotic association of AMF and plants is considered to be one of the most widespread plant-fungi associations, dating back to 460 million years, as per recent fossil evidence.^26^ An extensive and vast network of mycelia is formed by AMF that extends beneath the roots found across a wide range of plant species, creating the so-called common mycorrhizal network (CMN). The mycelial network formed can be divided into two groups. The first one is called the extra-radical mycelia (ERM), which connects the colonized root with the surrounding soil matrix and performs the primary function of uptake of water and nutrients like phosphorus from the soil and transporting it back to the plant. The second one is called the intraradical mycelia (IRM) network that penetrates deep into the root cortex, thus resulting in the formation of structures called arbuscules and vesicles that promote nutrient exchange and enhance storage, respectively.^27^ The CMN plays a significant role in terrestrial ecosystems, which influences plant communities as well as invasive species and facilitates nutrient transport, particularly phosphorus and nitrogen from fungi to plants.^28^ Various plant growth-related activities, such as the water potential of the leaf, stomatal conductance of the plant, relative water content (RWC), and assimilation of carbon dioxide, are also affected by the inoculation of AMF. As AMF successfully invades the host plant’s root epidermal cells, a symbiotic relationship is established. As per,^29^ the mycelial network synthesizes glycoproteins that help the host plant absorb and transport minerals and water, thereby forming a favorable rhizosphere environment.^27^ This significantly improves the ability of plants to absorb micro- and macro-nutrients and thus enhances their development. AMF can be used as biofertilizers and bioinoculants to increase crop production as a sustainable agricultural practice.^30^ They are known to improve soil structure, water-holding capacity (through substances like glomalin), and nutrient availability.^31,32^

AMF is found to enhance plant biomass accumulation by increasing the effective absorption range of plants with which it is associated in soil.^33,34^ AMF also facilitates long-distance nutrient exchange between the soil and plants, such as by polyphosphate translocation to the host plant through hyphae, utilizing specific aquaporins such as *Rhizophagus clarus* aquaporin 3.^35^ A membrane protein, namely MtPt, in *Medicago truncatula* phosphate transporter (MtPt) 4, present in the mycorrhizal roots, absorbs the inorganic phosphate released by AMF^36^ and has been demonstrated to be beneficial for plants and AMF symbiosis with plants.^37^ AMF induces tolerance to heavy metal toxicity to plants by releasing organic acids like citric acid, malic acid and forming complexes with the heavy metals through chelating and extracellular mycelium filtering processes. AMF provides stabilization and promotes detoxification of heavy metals through its different metabolic activities, which allow it to bind to the heavy metals and immobilize them. Due to this unique mechanism displayed by AMF, they have been considered a prime choice for the bioremediation of contaminated soil. ^38–40^

AMF and plant symbiosis exemplify a dynamic and intricate connection. AMF engages in interactions with other soil microorganisms, including plant growth-promoting rhizobacteria (PGPR). Such interactions have been observed in the case of heavy metal stress, as they pose a threat to microbial communities and plant growth. It has been shown that heavy metal-resistant PGPRs utilize bioaccumulation and biosorption strategies to limit metal uptake by plants. Bioaccumulation is an active process, while biosorption is a passive mechanism primarily taking place in the cell wall. To mitigate the heavy metal’s effect, AMF promotes the phytoremediation of heavy metals, resulting in an enhanced capacity of nutrient uptake in heavy metal-exposed plants. Thus, AMF helps restore soil health along with mitigating heavy metal phytotoxicity.^41^

AMF can contribute significantly to sustainable agriculture when used in organic farming techniques for plant growth and development. The symbiotic connection between AMF and plants is essential for the plant ecosystem and the viability of agriculture, as it encourages growth, improves nutrient absorption efficiency, and lowers dependency on synthetic fertilizers. Understanding and making use of the symbiotic connection can improve agricultural output, increase plant tolerance to environmental challenges, and facilitate more effective ecosystem restoration.

## 3. The AMF symbiotic relationship – the molecular aspects

The interactions between plant hosts and AMF are driven by a range of essential genes and signaling molecules that help establish and sustain this symbiotic relationship. The establishment and functioning of this symbiosis involve a variety of biomolecules and plant growth regulators (PGRs), involving the interactions of both fungal effector proteins and plant signaling pathways, which work together to facilitate nutrient exchange and mutual benefits. The key factors involved in the AMF symbiosis mechanism are as follows:

### 3.1. Mycorrhizal signalling molecules (Myc factors)

**Figure 2.**
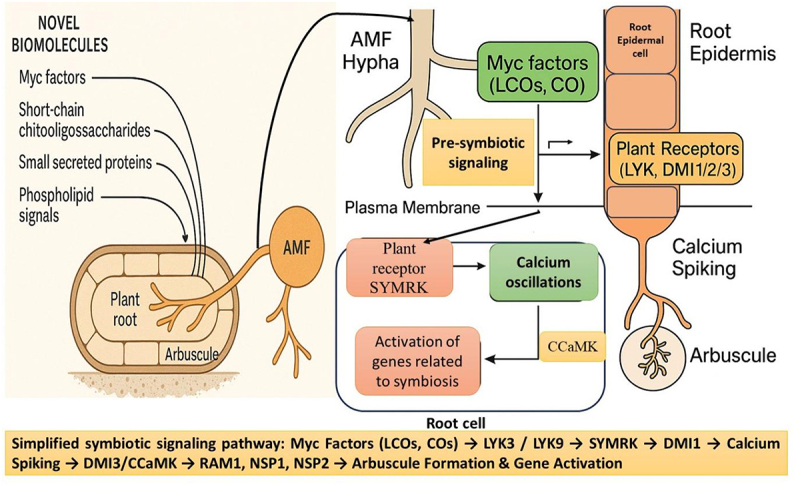
Molecular mechanism of AMF symbiotic relationship with the host plant. The diagram depicts how the myc factors facilitate the association. CCaMK – calcium and calmodulin-dependent protein kinase; CO – chitin oligomers; DMI – doesn’t make infection, LCO – lipochitooligosaccharides; LYK – lysine motif domain receptor-like kinase; NSP – nodulation signaling pathway; RAM – required for arbuscular mycorrhization; SYMRK – symbiosis receptor-like kinase.

During arbuscular mycorrhizal fungi (AMF) symbiosis, the root epidermis detects Myc factors, primarily lipochitooligosaccharides (LCOs) and short-chain chitin oligomers (COs), through specific receptor-like kinases (RLKs), initiating the common symbiosis signaling pathway (CSSP) (Figure 2).^12^ The initial recognition is mediated by LysM-domain RLKs such as LYK3, which perceives Myc factors and also functions in Nod factor signaling in legumes. LYK9/CERK (chitin elicitor receptor kinase) 1, has been demonstrated to play a dual role in distinguishing symbiotic and pathogenic fungi through chitin perception.^42,43^ Additionally, NFP (nod factor perception protein), though well-characterized in rhizobial symbiosis, also contributes to AMF recognition.^44^ Signal transduction involves co-receptors like SYMRK (Symbiosis receptor-like kinase)/DMI (Doesn’t Make Infection) 2, which collaborates with LysM-RLKs to propagate the signal to the nuclear envelope, activating DMI1, a cation channel responsible for initiating calcium spiking, a hallmark of symbiotic signaling.^45–47^ The oscillations are decoded by DMI3, also known as CCaMK (Calcium and Calmodulin-Dependent Protein Kinase), which activates downstream transcription factors such as RAM (required for arbuscular mycorrhization) 1, NSP (nodulation signaling pathway) 1, and NSP2, ultimately leading to arbuscule formation and expression of symbiosis-related genes.^48,49^ This coordinated molecular cascade ensures successful colonization and functional symbiosis (Figure 2).

The key fungal effector proteins are crucial for modifying host plant defenses and encouraging colonization, as observed in the case of *Funneliformis mosseae*. A recent study identified 120 effector proteins, significant for establishing AM interactions.^50^ Fungal effector proteins are essential molecular tools that facilitate the establishment and maintenance of symbiosis between arbuscular mycorrhizal fungi and plant roots. Pathogenic effector proteins suppress plant defense responses to facilitate infection, while arbuscular mycorrhizal fungi effector proteins behave as symbiosis-enhancing molecules to promote compatibility between the fungal partner and the host plant.^51,52^ AMF effector proteins also modify host gene expression, especially those involved in nutrient transport, symbiotic signaling, and cell wall remodeling, to support effective symbiosis.^53^ These effector proteins play a crucial role in the formation of arbuscules that facilitate nutrient exchange, enable effective phosphorus uptake by the plant and carbon delivery to the fungus and modulate plant growth regulator signaling pathways.^54,55^

### 3.2. Lipids and fatty acids

AMF has been known to alter the phospholipid signaling by modifying the concentrations of lysophosphatidylcholine (LPC) and lysophosphatidylethanolamine (LPE), which may serve as signaling molecules involved in regulating root architecture and cellular response.^56^ For example, SP (secreted protein) 7 effector protein secreted by *Rhizophagus irregularis*, which interacts with the ERF (ethylene response transcription factor) 19 transcription factor in the nucleus and suppresses ethylene-mediated defense pathways, eventually enhances fungal colonization.^51^ Collectively, these findings highlight the sophisticated role of AMF effector proteins in modulating host physiology to ensure the successful establishment of symbiosis.

The gateway of fatty acid translocation between the fungus and its host plants depends on the RAM (required for arbuscular mycorrhization) 2 gene, along with the ATP-binding cassette (ABC) transporter.^57^ The primary organic compound “lipid” is transferred from the host plant to AMF via the symbiotic connection.^58^ The RAM1 transcription factor is vital for AMF symbiosis, as it is responsible for the regulation of the activity of glycerol-3-phosphate acyltransferase, whereas RAM2 facilitates the transfer of lipids from plants to their associated AMF. The fatty acid thioesterase (Fat) M lipid biosynthetic enzyme and the ABC transporter (STR) are two important requirements for symbiosis in the early stage as they are uniquely conserved in plants that are involved in AMF symbiosis.^59^ The enhancer of root meristem (ERM) 1 and WRI (WRINKLED) 5a transcriptional activators bind to the promoters, leading to activation of the expression of genes such as STR, STR2 and FatM, which play important roles in fatty acid biosynthesis and transfer. An important regulatory role in arbuscular-containing cells is played by the ERM1/WRI5a-ERF12-TOPLESS (WRINKLED 5a, ethylene response factor 12) transcriptional regulatory complex, which helps to maintain a symbiotic relationship by maintaining stability and retaining benefits.^60^ AMF also derives sugars from the host plants. The SWEET (Sugar Will Eventually be Exported Transporter) family genes are also expressed and upregulated in arbuscular-containing cells.^61^

## 4. Involvement of AMF in imparting abiotic stress tolerance to plants

Abiotic stress refers to the negative impact of non-living factors on plants, these factors majorly include dehydration or drought stress, extreme low and high-temperature stress, and salt stress. These abiotic stresses significantly reduce agricultural output worldwide and impair plant growth and development.^60^ Several research studies have been performed to date in order to develop strategies to combat these stress conditions, which include physiological, biochemical, and molecular advancements.^62^ While these biotechnological developments offer accuracy and the potential to introduce new features, the AMF association offers a more organic, sustainable, and ecologically friendly method of enhancing resistance to abiotic stress. One significant evolutionary adaptation to the terrestrial environment would be the coevolution of mycorrhizae and plants. Arbuscular mycorrhizal (AM) association is a symbiotic association between fungi belonging to the phylum Glomeromycota with the roots of terrestrial plants. The symbiotic relationship enhances the host plant’s access to water and nutrients and as compensation, the fungus receives up to 20% of the carbon fixed by plants.^5^ Besides improving root functions and regulating plant responses, AMF can concurrently reduce the effect of abiotic stress factors by improving the efficiency of photosynthesis, increasing plant biomass, and regulating hormone function and signaling. Besides improving the plant’s performance under stress conditions, the association also enhances soil fertility, nutrient cycling, and carbon sequestration.^63^ Arbuscular mycorrhizal fungi (AMF) can thereby serve as bioinoculants that can symbiotically associate with a variety of agricultural plants and contribute to sustainable agriculture. The best approach to solving agricultural problems would be combining AMF with conventional breeding methods (Tables 1 and 2).

**Table 1.** Role of arbuscular mycorrhizal fungus (AMF) in abiotic stress tolerance. The table provides a summary of plant species and the corresponding AMF involved in abiotic stress tolerance. The plant species are listed alphabetically within each stress category.

Plant species	Effect on plant to mitigate stress	AMF Involved	References
Salinity stress			
*Cajanus cajan*	Enhanced functioning of enzymes such as SOD, CAT, DHAR, MDHAR, and GR	*Funneliformis mosseae* and *R. irregularis*	Pandey and Garg^64^
*C. annuum*	Increased levels of CAT, POX, APX enzymes. Enhanced activity of photosynthetic pigments, including chlorophyll a, chlorophyll b, and carotenoids.	*Glomus constrictum*	Al-Amri^65^
*Casuarina obesa*	Increased levels of CAT, SOD, and APX. Higher expression of proline and phenols. Increased nodule formation. Upregulation of Na-H anti-carrier transporter in roots.	*Rhizophagus fasciculatus*	Djighaly et al.^66^
*Cocos nucifera*	Increases in glycoprotein (glomalin) production by extramatrical mycelia. Increases in the availability of essential nutrients such as N, K, and P in the rhizosphere.	*Glomus sp*., *Funneliformis sp.*, *Acaulospora sp*., *Gigaspora sp*., and *Scutellospora sp*.	Sulistiono et al.^67^
*Crocus sativus*	Increases total chlorophyll, protein, proline, and betaine glycine content, as well as antioxidant activity. Induced expression of metallothionein, Cu/Zn, SOD. Aquaporin (AQP) activation.		El Aymani et al.^68^
*Gossypium hirsutum*	Enhanced activity of antioxidant enzymes (SOD, POD, CAT). Increase in chlorophyll levels. Accumulation of root endogenous hormones like IAA.	*Paraglomus occultum*	Zhang et al.^69^
*Linum usitatissimum*	Increased chlorophyll content. Increased nitrogen and phosphorus content. Increased salt concentration (NaCl).	*Claroideoglomus etunicatum*, *F. mosseae*, *Glomus aggregatum*, and *Rhizophagus intraradices*	Kakabouki et al.^70^
*Musa spp.*	Induction of stress-responsive genes.	*Acaulospora scrobiculata* and *Glomus clarum*	Rashad et al.^71^ Yano-Melo, Saggin and Maia^72^
*Narcissus spp.*	Improved regulation of ion homeostasis and osmotic balance, along with increased antioxidant enzyme activity. Notable interactions between salt and mycorrhiza were observed in bulbs for N, P and Fe in roots and for P, Ca, Mg, Fe, Mn, and Cu in leaves.		Çığ, Gülser and Gülser^73^
*O. sativa*	Increases in osmotic regulators like soluble sugars, proline, polyamines, and betaine.	*Funnelliformis mosseae* *Acaulospora laevis* and *Gigaspora margarita*	Mitra et al.^74^; Parvin et al.^75^
*Satureja khuzistanica*	Increase in chlorophyll, sodium-potassium and, proline content. Increase in antioxidant enzymes.	*Glomus intraradices*	Saadatfar, Hossein and Jafari^76^
*Sorghum bicolor*	Improved mineral nutrition. Ensuring elevated levels of K^+^/Na^+^ ratio with a reduction in shoot/root Na^+^ ratio. Modified osmotic adjustment by accumulating soluble sugars.	*Acaulospora mellea*	Wang, Sun and Shi^77^
*Stevia rebaudiana*	Increase in antioxidant enzymes like POD, SOD, and CAT. Higher expression of Chlorophyll a	*R. irregularis*	Janah et al.^78^
*Tulipa spp.*	Induction of significant changes in plant root morphology. Promoting root growth by high branching of plant roots and formation of higher secondary roots and fine roots.		Bilias et al.^79^
**Dehydration/Osmotic stress**			
*Allium ampeloprasum*	Fungal mycelium increases the total absorption area thereby improving access to immobile elements such as P, Cu, and Zn.	*Funneliformis geosporum*	Hibilik et al.^80^; Karima et al.^81^
*A. sativum*	Increase in nutrient and water absorption. Improvement in seedling survival, bulb development and thereby higher onion yield.	*Funneliformis mossesae* and *C. etunicatum*	Patale^82^ Muhsen, Hameid and Al-Attabi^83^
*Avena sativa*	Upregulation of genes related to IAA synthesis. Increase in root length and root density	*R. intraradices*	Tian et al.^84^ Zhang et al.^85^
*Beta vulgaris*	Elevated activity of antioxidant enzymes such as CAT and APX. Increased proline content and photosynthetic pigments.	*F. mosseae*, *A. laevis*, and *Gigaspora gigantea*	Yadav et al.^86^
*Curcuma longa*	Enhanced fluorescein diacetate dehydrogenase and alkaline phosphatase (DHA and FDA) activity in the soil. Increase in the total chlorophyll, carotenoid content and stomatal conductance.		Jabborova et al.^87^
*G. max*	Increased chlorophyll metabolism Increased activity of nitrate reductase, superoxide dismutase, and catalase.	*R. irregularis*	Begum et al.^88^
*Helianthus annuus*	Increased chlorophyll content and mineral uptake. Enhanced synthesis of plant hormones like auxin, cytokinin, and gibberellins.	*Glomus mosseae*	Sharma, Delta and Kaushik^89^
*Lactuca sativa*	Increased proline accumulation. Increase in polyphenols content, sodium, potassium, and calcium content. Increased production of phytohormones.	*R. irregularis*	Abdel-Ilah et al.^90^; Kojić et al.^91^
*Lallemantia spp.*	Increase in chlorophyll concentration. Increased nutrient uptake.		Paravar, Farahani and Rezazadeh^92^
*Medicago sativa*	Induces proline accumulation	*R. intraradices*	Bahadur et al.^93^
*Prunus persica*	Increased proline accumulation.	*G. mosseae*	Zheng et al.^94^
*Quercus robur*	Increased concentration of carbon and nitrogen content in leaves. Increase in antioxidant enzymes like CAT, SOD, and POD. Upregulation of proline metabolizing genes. Accumulation of higher levels of polyamines (spermine and spermidine) and 12-oxo-phytodienoic acid.		Kebert et al.^95^
*Saccharum spp.*	Increase in total chlorophyll and carotenoid content. Promotes the expression of genes and protein transporters that include AQPs, inorganic phosphorus transporters, and ammonium, nitrate, sulfur, zinc, and carbon transporters.	*G. intraradices*	Spinoso-Castillo et al.^96^
*Solanum tuberosum*	Increase in phenolic compounds, antioxidant enzymes, and photosynthetic pigments.	*Claroideoglomus claroideum* and *Claroideoglomus lamellosum*	Nahuelcura et al.^97^
*Z. mays*	Enhanced nitrate reductase activity. Optimization of H2O2 levels and ROS concentrations.	*Glomus versiforme*	Begum et al.^98^
**Low-temperature stress**			
*H. vulgare*	Accumulation of soluble sugars and proline.	*G. versiforme* and *R. irregularis*	Joudmand, Hajiboland and Aliasgharzad^99^
*S. lycopersicum*	Increased levels of SOD, POD, and APX enzymes. Increase in proline accumulation.	*F. mosseae* and *Septoglomus constrictum*	Chandrasekaran, Boopathi & Manivannan^100^
**High-temperature stress**			
*A. officinalis*	Antioxidant enzyme activity, including superoxide dismutase and ascorbate peroxidase, is elevated.	*G. intraradices*	Yeasmin et al.^101^
*C. sativus*	Increased accumulation of soluble sugars, proline, and betaine. Increased expression of Hsp genes.	*Diversispora versiformis*	Liu et al.^102^
**Heavy metal stress**			
*C. cajan*	Activation of H±ATPase for nutrient and water uptake. Increased chlorophyll a/chlorophyll b ratio.	*R. irregularis*	Garg and Kashyap^103^
*Lavandula angustifolia*	Increase in proline content and soluble sugars. Increased antioxidant enzyme activity.	*F. mosseae*	Rasouli et al.^104^
*Iris spp.*	Increased abundance of heavy metal resistance genes and nutrient cycling genes. Increased secretion of ferric carriers, indole acetic acid, and hydrocyanic acid, along with organic acids and biosurfactants, to reduce heavy metal toxicity.		Zhao et al.^105^
*Pisum sativum*	Increase in chlorophyll concentration. Accumulation of proline. Increased levels of CAT, SOD, and APX enzymes.	*G. mosseae*	Chaturvedi et al.^106^
*T. aestivum*	Promotes nutrient balance. Reduces membrane permeability. Boosts photosynthetic pigments. Increases sugar accumulation. Inhibits MDA (malondialdehyde) synthesis.	*A. laevis*, *F. geosporum*, *F. mosseae*, and *Cetraspora armeniaca*	Farghaly, Nafady and Abdel-Wahab^107^
*Multiple stressors - Salinity and Osmotic/dehydration stress			
*Agave tequilana*	Promotion of plant growth through growth-regulating substances, which enhances water absorption and nutrient uptake (especially P, Cu, Zn, N, etc.). Improved hydraulic conductivity in roots and increased CO_2_ absorption.		Montoya-Martínez et al.^108^
*Ananas comosus*	Enhanced activity of antioxidants – superoxide dismutase (SOD), catalase (CAT), glutathione reductase (GR), peroxidase (POX), and polyphenol oxidase (PPO)		Moreira et al.^109^
*Metroxylon sagu*	Improvement in the uptake of plant nutrients – phosphorus (P) and nitrogen (N).	*Acaulospora sp*. and *Glomus sp.*	Asano et al.^110^
*Panicum miliaceum*	Improvement in nutrient uptake, especially phosphorus (P). Increased accumulation of soluble sugars in the roots. Adjustment of K^+^/Na^+^ ratio. Increased antioxidant enzymatic activities.	*Piriformospora indica*	Caruso et al.^111^
*Petroselinum crispum*	Increased levels of proline, soluble sugars, polyamines, and sodium and potassium ions. Increased antioxidant concentrations like SOD, POD, and APX.	*R. irregularis* and *C. etunicatum*	Israel et al.^112^
*Phoenix dactylifera*	Maintenance of high-level of leaf water parameters in the plant. Advanced water parameters including stomatal conductance provide increased tolerance to the stress.	*Glomus monosporus*, *G. clarum*, and *Glomus deserticola*	Meddich et al.^113^
*Multiple stressors -*Salinity, extreme temperature and dehydration stress*			
*Arachis hypogaea*	Increased chlorophyll content and antioxidant enzymes. Significant increase in Sodium: Potassium Ratio	*R. irregularis*, *R. clarus*, *Glomus lamellosum*, and *F. mosseae*	Liu et al.^114^
*Vigna spp.*	Increased antioxidant enzymes. Higher proline level. Increased micronutrients and macronutrients in plants.		Loo et al.^115^

*denotes the involvement of AMF in multi-stress tolerance.

**Table 2.** Genes of the host plant involved in arbuscular mycorrhizal fungus-mediated abiotic stress tolerance.

Host Plant	AMF associated with the host plant	Host plant Gene Acronym	Gene name	Gene regulation upon abiotic stress conditions	Reference
*Casuarina glauca*	*R. irregularis* *G. mosseae*	*HAK5*	High-affinity K+ transporter	*HAK5* is upregulated in *C. glauca* under salinity stress.	Wang, Dong and Tang^116^
		*KAT3*	K+ affinity transporter	*KAT3* is upregulated in *C. glauca* under salinity stress.	Huertas et al.^117^
		*SKOR*	Shaker-type K+ outward rectifier	*SKOR* is upregulated in *C. glauca* under salinity stress.	Yaish et al.^118^
		*CPER*	Putative cytochrome c peroxidase	*CPER* is upregulated in *C. glauca* under salinity stress.	
*C. sativus*	*P. occultum*	*CsHsp70*	Heat Shock Protein 70	*Hsp70* is upregulated in *Cucumis sativa* under waterlogging stress.	Xiang et al.^119^
		*CsPIP*	Plasma membrane Intrinsic Protein	*PIP*, a plasma membrane intrinsic protein, is upregulated in *Cucumis sativa* under waterlogging stress.	Land et al.^120^; Afzal et al.^121^
*G. max*	*G. clarum*	*P5CS*	Pyrroline-5-carboxylate synthetase	Downregulation of the *P5CS* gene in drought-stressed conditions.	Sheteiwy et al.^122^
	*G. margarita*	*PDH*	Proline dehydrogenase	Downregulation of the *PDH* gene under drought stress.	
	*G. mosseae*	*P5CR*	Pyrroline-5-carboxylate reductase	Downregulation of the *P5CR* gene under drought stress.	
*P. miliaceum*	*G. mosseae*	*PaMT1, PaMT2* and *PaMT3*	Methyltransferase	*PaMT* is upregulated in *P. miliaceum* under heavy metal stress.	Cicatelli et al.^123^
		*PaSPDS*	Spermidine Synthase	*PaSPDS* is upregulated in *P. miliaceum* under heavy metal stress.	Sharma et al.^124^
		*PaADC*	Arginine Decarboxylase	*PaADC* is upregulated in *P. miliaceum* under heavy metal stress.	
*P. trifoliata*	*G. mosseae*	*CSD1*	Copper/Zinc SOD	*CSD1* is upregulated in *Poncirus trifoliata* under salinity stress.	Fan et al.^125^
	*Trifolium repens*	*MIOX1*	Myo-inositol oxygenase	*MIOX1* is upregulated in *P. trifoliata* under salinity stress.	
		*GLX1*	Glyoxalase I	*GLX1* is upregulated in *P. trifoliata* under salinity stress.	
		*TT5*	Transparent Testa 5	*TTC5* is upregulated in *P. trifoliata* under salinity stress.	
*Musa acuminata*	*Clomus clarum*	*JERF3*	Jasmonate and ethylene-responsive Factor 3	Expression of the gene *JERF3* is upregulated under salinity stress.	Rashad et al.^126^
	*Rhizoglomus clarum*	*POD*	Peroxidase	Expression of the gene *POD* is upregulated under salinity stress.	Pegoraro et al.^127^
	*F. mosseae*	*PR1*	Pathogenesis-related protein 1	Expression of the gene *PR1* is upregulated under salinity stress.	Zheng et al.^128^
		*GLU*	Glucanase	Expression of the gene *GLU* is upregulated under salinity stress.	Gupta et al.^129^
		*CHI*	Chitanase	Expression of the gene *CHI* is upregulated under salinity stress.	
		*CsHsp70*	Heat Shock Protein 70	*Hsp70* is upregulated in *Cucumis sativa* under waterlogging stress.	Zeng et al.^130^
*S. lycopersicum*	*F. mosseae*	*Pht*	Phosphate transporter	Genes are upregulated under osmotic stress and heat stress conditions.	Szentpéteri et al.^131^
		*Pip*	Plasma intrinsic protein	Upregulation of *PIP2.5* gene expression and downregulation of *PIP2.7* gene expression.	
		*HSP*	Heat shock protein	Downregulation in the expression of the *HSP70* gene in plants after mycorrhizal inoculation.	
*O. sativa*	*F. mosseae*	*OsQHB*	*O. sativa* quantitative trait locus for heading date and branching	Upregulation of genes in AMF-inoculated plants enhances salt stress tolerance.	Zhang et al.^132^
		*OsPRX2* *OsPRX4* *OsPRX9* *OsPRX72* *OsPRX112* *OsPRX-A2*	*O. sativa* Peroxidase	Upregulation of genes in plants inoculated with AMF enhancing salt stress tolerance.	
		*OsABSRP5*	*O. sativa* ABC transporter Protein 5	Upregulation of genes in plants inoculated with AMF enhancing salt stress tolerance.	
*Z. mays*	*Funneliformis constrictum*	*ADC*	Arginine decarboxylase	Upregulation of *ADC* gene in plants inoculated with AMF under salinity stress.	El-Sawah et al.^133^
		*DAO*	Diamine oxidase	Upregulation of *DAO* activity in shoots of AMF- inoculated plants in saline stress	El-Sawah et al.^133^
		*POA*	Polyamine oxidase	Downregulation of *PAO* gene in AMF-associated plants in saline stress	Gao et al.^134^
		*ODC*	Ornithine decarboxylase	Upregulation of *ODC* gene in plants inoculated with AMF under salinity stress.	Sheteiwy et al.^135^

### 4.1. Salinity stress

Saline stress poses significant challenges to plant growth, particularly in irrigated lands, impacting agricultural industries worldwide. Approximately one-fifth of the land irrigated worldwide is harmed by salinization.^136^ The problem of salinization is growing, further complicating agricultural practices and plant distribution. Saline stress reduces water availability for plants as sodium ions (Na^+^) accumulate around roots. Sodium (Na^+^) and chloride ions (Cl^−^) exert toxic effects on plants, disrupting essential plant processes.

Several defense strategies are employed by plants to cope with saline stress, involving complex signaling pathways and symbiotic relationships with beneficial microorganisms such as AMF. To preserve osmotic equilibrium and maintain cellular structures, plants generate suitable solutes such as proline and glycine betaine. Active transport mechanisms have been developed to remove toxic ions (e.g., Na^+^) from cells or compartmentalize them in vacuoles to reduce their harmful effects. Plants regulate ROS levels through enzymatic and non-enzymatic antioxidants to prevent oxidative damage. Plants adjust their growth rates under saline stress to allocate resources efficiently and prioritize stress tolerance mechanisms. The symbiotic relationship with AMF has been proven instrumental in enhancing saline stress tolerance by improving nutrient uptake (especially phosphorus), enhancing water uptake, and modulating plant hormone levels.^137,138^ Understanding the mechanisms behind AMF-mediated saline stress tolerance is very important for developing techniques for reducing the adverse effects on crops. Effective management practices and genetic improvements in crop varieties tolerant to saline stress are essential for sustaining agricultural productivity in affected regions.

Under saline stress conditions, mycorrhizal associations have a significant impact on enhancing plant adaptation and mitigating damage. A transcriptome analysis revealed 391 differently expressed transcripts regulated by AMF in the seedling roots of *Asparagus officinalis* under saline stress. These transcripts primarily belong to ROS homeostasis, cell wall construction, mineral elements, and water uptake regulation.^139^ Mycorrhizal halophytes have been shown to exhibit lower concentrations of toxic sodium ions (Na^+^) compared to non-mycorrhizal halophytes under saline stress conditions. Differences in soluble sugar concentrations suggest that mycorrhizal associations influence carbohydrate and energy metabolism, potentially through pathways involving glyoxylate as well as dicarboxylic acid metabolism.^140^ Further, AMF has been demonstrated to improve chlorophyll content, photosynthetic rates, and fluorescence parameters in leaves of salt-stress-induced plants, leading to enhanced utilization of light energy, contributing to improved plant growth and reduced salt-inflicted damages.^136^ AMF symbiosis has also been demonstrated to maintain osmotic equilibrium by altering Na^+^/K^+^ balance in plants. This adjustment is crucial for managing osmotic stress and ensuring proper cellular function.^27^

In the *Ziziphus jujuba* roots, the AMF colonization has demonstrated the enhancement in root H^+^ efflux and K^+^ influx under saline stress. This is achieved by AMF-dependent activation of *ZjAHA* (*Z. jujuba* autoinhibited H^+^ -ATPase) *7*, a plasma membrane ATPase responsible for H^+^ efflux from cells. The expression of *ZjHAK* (*Z. jujuba* Autoinhibited H^+^ -ATPase) *2*, likely a potassium transporter gene, is also increased by AMF colonization. This results in K^+^ accumulation within cells, leading to a high K^+^/Na^+^ quantity that helps mitigate salt stress effects. This helps in maintaining ion balance and cellular homeostasis. AMF symbiosis has also been observed to increase the fatty acid metabolism in the leaves and roots region mostly, of jujube plants under saline stress. Higher fatty acid content contributes to enhanced salt tolerance.^141^

It is also observed that AMF colonization leads to an upregulation of genes like *OsPRX (Oryza sativa* peroxidase), *Os10g (O. sativa* chromosome 10 gene), *OsHBP1b* (*O. sativa* heptahelical binding protein 1b), and *OsNCX* (*O. sativa* sodium/calcium exchanger) in *O. sativa* under saline-stress conditions. This enhanced gene expression has helped improve ROS scavenging ability and reduced malondialdehyde (MDA) accumulation in mycorrhizal plants, indicating lower oxidative stress levels.^27^

AMF, *Rhizopus irregularis* and host plant *Casuarina glauca* association studies have demonstrated the increased expression of ion transport genes (*HAK5;* high-affinity K^+^ transporter 5, *PIP1-2;* plasma membrane intrinsic protein 1–2), ROS scavenging (*MYB;* myeloblastosis transcription factor 46, *NAC43;* NAM, ATAF1/2, CUC2 transcription factor 43), and carbohydrate metabolism (*GLP10;* Germin-like protein 10, *SKOR;* stelar K^+^ outward rectifier, *CPER;* cation proton exchanger-related, *WRKY19;* WRKY transcription factor 19) genes under saline-stress conditions. These genetic enhancements contribute to increased saline stress tolerance in mycorrhizal plants, potentially by improving ion balance, osmotic adjustment, and stress response pathways.^142^ In addition to the Na^+^ and Cl^−^ ion compartmentalization in vacuoles, which increases saline stress tolerance, the increased expression of *CgNHXs* (*C*. *glauca* Na^+^/H^+^ exchangers) and *CgCLCs* (*C. glauca* chloride channel proteins) has resulted in higher biomass and potassium content.^141^ In *Robinia pseudoacacia*, AMF association led to enhanced expression of *SOS1*/*NHX7* (salt overly sensitive 1/Na^+^/H^+^ exchanger 7), leading to saline stress tolerance by effluxing Na^+^ out of plant roots.^143,144^

AMF colonization has been demonstrated to upregulate the expression of salt-sensitive SOS (salt overly sensitive) 1 and 2 genes of *Solanum lycopersicum* under saline stress conditions. This up-regulation improves the saline tolerance of *S*. *lycopersicum*, potentially by improving sodium ion (Na^+^) efflux and maintaining ion homeostasis in cells.^143^ In *Sesbania cannabina*, the AMF inoculation altered hub gene expression related to response against saline stress. This includes transcription factors belonging to the families of the *WRKY* (WRKYGQK domain-binding protein), *ERF* (Ethylene Response Factor), *MYB*, and *TCP* (TEOSINTE BRANCHED1, CYCLOIDEA, and PCF (Proliferating Cell Factor)), which play crucial roles in regulating stress-responsive genes and pathways.^145^

There have also been reports that AMF inoculation improves the fresh and dry weight of plants, along with nitrogen concentration in shoots and roots, under moderate saline conditions.^22^ In *Antirrhinum majus*, AMF has been shown to significantly enhance the growth rate of plants, water potential of leaves, water utilization efficiency,^146^ enhanced photosynthetic rate, and stomatal conductance under saline regimes.^147^ Similarly, in *Allium sativum*, the AMF-inoculated plants showed enhanced growth characteristics, along with increased leaf area index and fresh and dry biomass under saline conditions.^146,148^ In *Ocimum basilicum*, AMF association under conditions of salinity stress showed improved photosynthetic rate paired with increased chlorophyll content and gas exchange traits.^149^

Plants in association with AMF also showed a higher accumulation of ions and elements, such as total content of P, Ca^2+^, N, Mg^2 +^, and K^+^, compared to uninoculated plants in salt stress conditions, as demonstrated in the case of *Cucumis sativus* ^150^ and *Capsicum annuum*.^151^ AMF inoculation also exhibited higher biomass production, increased proline synthesis, improved N uptake, and a decrease in Na^+^ content in plant cells compared to plants not having mycorrhizal association under stress, as shown in the case of lettuce plants.^152^ AMF inoculation also improved cytokinin concentration, enhancing photosynthate translocation under salt stress,^153,154^ Alterations in the polyamine pool are also linked with AMF-mediated growth promotion.^155^

These studies contribute to our understanding of how AMF modulates responses by plants toward salinity stress through genetic as well as regulatory mechanisms. The activation of key genes and transcription factors by AMF colonization helps plants cope with this stress by improving ion transport, osmotic adjustment, and stress response pathways. The findings underscore the beneficial effects of AMF in improving saline stress tolerance in plants, using mechanisms like ion regulation, carbohydrate metabolism, and photosynthetic efficiency. By facilitating these adaptations, mycorrhizal associations contribute to the overall resilience of halophytes and other salt-stressed plants, promoting their growth and survival in saline environments.

### 4.2. Dehydration stress

Dehydration stress significantly impacts plant life by limiting water availability, reducing transpiration rates, and inducing stress by oxidation.^156,157^ These conditions negatively affect the growth of plants by diminishing enzyme activity, ion uptake, and nutrient assimilation.^158,159^ Recently, AMF has been proven to improve dehydration stress in various crop plants, including *Triticum aestivum* (wheat), *Hordeum vulgare* (barley), *Zea mays* (maize), *Glycine max* (soybean), *Fragaria ananassa* (strawberry), and *Allium cepa* (onion). ^160–163^

AMF colonization encourages the development of the fungi’s extra-radical hyphae in the plant roots, which allow plants to explore a greater volume of soil. This extensive network allows for more efficient water and nutrient uptake, which is critical during drought conditions. ^164–167^ AMF has been demonstrated to enhance plant’s water relations using enhanced root hydraulic conductivity and maintaining higher leaf water potential. This results in better water retention along with reduced wilting under circumstances of drought.^162^ AMF has also been demonstrated to mitigate oxidative stress by enhancing the antioxidant defense system of plants. It increases the activity of various antioxidant enzymes, such as superoxide dismutase (SOD), catalase (CAT), and peroxidase (POD), that help in scavenging ROS generated during circumstances of drought.^157^

AMF symbiosis increased rates of transpiration, stomatal conductance, gas exchange, and water relations in the leaf.^161,168^ These improvements are crucial for maintaining photosynthesis and plant health during stress. AMF uses abscisic acid (ABA) responses to regulate stomatal conductance and other associated physiological processes.^169^ By modulating stomatal behavior, AMF helps plants to conserve water and maintain cellular turgor under drought stress. Recent studies also show that both C3 (*Leymus chinensis*) and C4 (*L. chinensis*) plants exhibit AMF-mediated enhancement in growth and photosynthesis under drought-mediated stress through the increased expression of the antioxidant system.^170^ This enhancement is vital for sustaining plant productivity under adverse environmental conditions.

AMF inoculation in *T*. *aestivum* has been shown to enhance dehydration tolerance by improving water uptake along with increased antioxidant enzyme activities.^162^ In *H. vulgare*, the AMF association improved dehydration stress tolerance by enhancing root growth and better water relations.^161^ The dehydration stress tolerance in *Z*. *mays* due to AMF association occurred due to increasing root hydraulic conductivity and maintaining higher leaf water potential.^163^ AMF-improved nutrient uptake has been demonstrated to enhance the antioxidant defense mechanisms in *G. max*, leading to dehydration stress tolerance.^160^

### 4.3. Extreme temperature stress

The increase in global mean temperature has resulted in temperature variability, causing plants to face temperature extremes that are different from their optimal growth range.^171^ Two of the major temperature-dependent abiotic stresses that have led to major food security issues across the globe are heat and cold stress.^172^ To address this issue, one promising and eco-friendly approach is the AMF-induced stress tolerance.^173^ Some of the major changes observed in heat-stressed plants, upon AMF inoculation are an increase in chlorophyll content, a significant decrease in H_2_O_2_ and MDA content, which are the significant markers for stress.^174,175^

*C. sativus* has an ambient growth temperature of 30°C/15°C, day/night temperature, but when the temperature exceeds 35°C it results in a change of morphological characteristics such as reduction in plant growth and wilting of stems and leaves, eventually leading to the death of the plant. Upon AMF inoculation, two stress-responsive genes (plasma membrane intrinsic proteins (*PIP*) and heat shock protein (*Hsp*) showed increased expression, helping in mitigating the stress response and protecting the plant from cellular and oxidative damage.^102^ AMF inoculation to perennial ryegrass (*Lolium perenne*) improved plant growth when subjected to high-temperature stress resulting in an increase in the antioxidant enzyme activity such as SOD), POD, CAT and ascorbate peroxidase (APX) in the roots of *L*. *perenne*, which are primarily responsible scavenging ROS and protecting plants from damage due to oxidative stress.^176^

Cold stress is another major issue that is responsible for the reduction of plant productivity, exacerbating the problems with the food supply in the face of a growing global population. Cold stress conditions increase the biosynthesis of various phytohormones such as gibberellic acid (GA), salicylic acid (SA), ethylene (ET), jasmonic acid (JA), auxin (IAA), cytokinin (CK), and abscisic acid (ABA), helping plants survive under cold stress conditions.^177^ Four genes necessary in JA biosynthesis, named as LOX1, LOX2, AOC, and JAR1, have been found to be upregulated under cold stress conditions.^178^ AMF has been therefore known to improve stress tolerance in *S. lycopersicum* by reducing the level of MDA, H_2_O_2_, and O^2-^.^15^ AMF employs several mechanisms in crops like *Z*. *mays*, *O*. *sativa*, *S*. *lycopersicum*, and *Cucumis melo*, including enhanced photosynthetic efficiency, improved respiration, increased nutrient uptake, osmotic adjustment, control of root hydraulic conductance, effective scavenging of reactive oxygen species (ROS), and transcriptomic-level adaptations. ^179–182^

### 4.4. Heavy metal toxicity

Heavy metal pollution poses significant threats to plants due to its detrimental effects on cellular processes and enzyme activities. Heavy metals like cadmium (Cd) disrupt ion balance within plants, leading to metabolic disorders and impairing normal physiological activities. Cd can also form complexes with proteins and enzymes, displacing essential elements and causing functional impairment, inactivation, or denaturation of these biomolecules.^183^ Plants have developed various mechanisms to counter heavy metal toxicity, such as plants undergoing various responses to mitigate Cd-induced damage, including changes in antioxidant defense systems to manage reactive oxygen species (ROS) bursts triggered by Cd stress. These adaptations help plants cope with oxidative stress and maintain cellular functions under Cd contamination.^184^ Recently, the usage of AMF has proven to be beneficial against heavy metal toxicity. Heavy metal tolerance is improved by enhancing nutrient uptake (e.g., phosphorus), facilitating detoxification processes, and modulating antioxidant systems to counteract oxidative stress induced by heavy metals, such as cadmium (Cd), uranium (U), zinc (Zn), copper (Cu) and lead (Pb). ^185–188^ These results demonstrate the possibility of AM symbiosis in mitigating the detrimental consequences of heavy metal contamination on plants. Understanding these mechanisms is an important aspect of developing sustainable agricultural practices and remediation strategies to tolerate the effects of contaminants on ecosystem stability and plant health.

The AMF provided tolerance toward heavy metals mainly via two mechanisms, like the growth dilution effect along with mycorrhizal immobilization. AMF can enhance phosphorus (P) uptake efficiency in plants, leading to increased biomass accumulation. As a result, the concentration of heavy metals in plant tissues is reduced, reducing their phytotoxic effects. By improving nutrient availability (especially P), AMF supports overall plant growth and vigor, which can reduce the impact of heavy metals on plant health.^189,190^ Under the mycorrhizal immobilization method, AMF immobilizes heavy metals within plant roots through the formation of polyphosphate complexes. This sequestration inhibits the heavy metals’ translocation from roots to shoots, thereby reducing their accumulation in above-ground plant parts. By limiting the movement of heavy metals into shoots, AMF helps mitigate their harmful impacts on the cellular functions and metabolism of plants.^191^

Along with these, AMF facilitates the methylation processes in plants exposed to heavy metals. Methylation is a biochemical process that modifies the chemical form of metals, potentially reducing their toxicity to plants.^192^ By promoting methylation, AMF converts heavy metals into less toxic forms or facilitates their sequestration, thereby protecting plants from phytotoxic effects.^191^ Further, AMF secretes compounds such as lipids and proteins that enhance antioxidant enzyme activities in plants. These enzymes, like superoxide dismutase and catalase, help in the scavenging of reactive oxygen species (ROS) generated under heavy metal stress. They may also modulate the expression of genes involved in heavy metal stress responses by plants. By influencing gene expression, AMF helps plants to counter oxidative stress and other damage due to heavy metals.^193^

These results highlight AMF’s diverse function in improving plants’ resistance to stress and heavy metal toxicity. By facilitating methylation processes, improving antioxidant defenses, and modulating gene expression, AMF contributes significantly to mitigating the adverse impacts of heavy metals on the development and growth of plants. Understanding these mechanisms can inform strategies for sustainable agriculture along with environmental management practices in contaminated areas.

In *M. truncatula*, it has been observed that AMF downregulates respiratory burst oxidase homologs (RBOHs) gene expression, specifically MtRbohC-G, under Pb stress. This down-regulation decreases the accumulation of hydrogen peroxide (H₂O₂), helping maintain ROS homeostasis and along with oxidative stress in the plants.^194^ AMF induces the synthesis of essential plant hormones, including gibberellins (GA), abscisic acid (ABA), and indole-3-acetic acid (IAA) in host plants under heavy metal stress. These hormones play crucial roles in enhancing plant resistance to such stress. In maize plants, AMF symbiosis has been demonstrated to result in differential hormonal responses under Cd toxicity, depending upon the type of maize cultivar used. High Cd uptake cultivars led to an increase in ABA levels, which likely contributed to stress tolerance mechanisms such as stomatal closure and stress signaling. On the contrary, the low Cd uptake cultivar increases IAA levels, which may promote growth and development of roots, enhancing the plant’s ability to cope with Cd stress.^195^ In the *Malus pumila* (apple) plant, the silencing of *MdGH3–2/12* has been related to reduced Cd stress and comparatively lower AMF symbiosis ratio as compared to the wild-type plant. This suggests that to counter Cd stress, these genes are very important in mediating the interaction between AMF and host plants.^196^ These findings highlight the complex interactions between AMF and host plants in response to heavy metal stress, involving both ROS management and hormonal regulation. By modulating gene expression and hormone synthesis, AMF helps plants by enhancing overall stress tolerance and promoting healthier growth under stress due to heavy metals. Understanding these interactions can inform the development of more effective strategies for managing heavy metal pollution in agricultural systems.

Involvement of heavy metal ATPase (HMA) genes in copper (Cu) toxicity under the influence of AMF symbiosis has also been observed. In maize plants, the AMF association leads to the upregulation of HMA genes, *ZmHMA3* and *ZmHMA4* isoforms, which are believed to encode proteins involved in Cu detoxification, suggesting that their upregulation is a response mechanism to mitigate Cu toxicity in the plants. By inducing the expression of *HMA* genes, AM symbiosis likely improves the plant’s capacity to detoxify and manage excess Cu, thereby reducing its toxic effects. Two Cu transporter (CTR) family members (*RiCTR1* and *RiCTR2*) and a CTR-like protein (RiCTR3A) have been identified in the AM fungus, *R. irregularis*. These genes are involved in Cu transport and tolerance.^197^ The latter one, RiCTR3A, acts as a Cu receptor in Cu stress conditions, helping to manage Cu levels within the fungus. Further, three additional genes were identified, where *RiATOX1* encodes a putative chaperone that mediates the Cu transfer to ATPases; *RiSco1* encodes a chaperone involved in transportation of Cu to cytochrome C oxidases; and RiSSC encodes a Cu or Zn superoxide dismutase chaperone.^198^

## 5. Involvement of plant hormones in AMF-mediated abiotic stress tolerance

Various changes in abiotic factors lead to stress conditions in plants and result in a detrimental impact on the quality and yield of plant products, and also deteriorate growth in plants. To counter this, plants biosynthesize plant growth regulators (PGRs) referred to as plant hormones or phytohormones, which are auxins, gibberellins, cytokinins, salicylic acid (SA), jasmonic acid (JA), brassinosteroids, polyamines (PA), strigolactones (SL), and nitric oxide (NO). These phytohormones help plants mitigate stress as well as play a crucial role in establishing plant-microbe interactions.^199^

### 5.1. Auxin and cytokinin

Phytohormones play a crucial role in various aspects of plant life, including AMF-plant interaction as well. ^135,199,200^ An increase in concentration of IAA has been observed in the roots of soybeans inoculated with AMF, as compared to the uninoculated plants. This increase in IAA promotes the development of roots, particularly lateral roots.^200^ Increased levels of IAA due to AMF influence have also been linked to the proliferation of lateral roots.^201^ Altered levels of IAA and abscisic acid (ABA) phytohormones have been observed in signaling in jujuba roots, under the effect of AMF. However, these levels are reduced under salt stress conditions, indicating a complex interaction between AMF symbiosis, hormone levels, and stress responses.^202^ The increase in IAA levels due to AMF inoculation can be attributed to the role of auxin in enhancing root structure, which can improve uptake of nutrients and, resulting in overall plant growth. Further, the modulation of IAA and ABA levels as a result of AM symbiosis and environmental stresses such as salinity underscores the importance of these hormones in adapting to challenging conditions. Hormonal imbalances caused by root damage have been observed to be reversed by AMF by increasing the levels of phytohormones IAA and cytokinin while decreasing the ABA levels in roots and leaves.^139^ The triggering of hormone signaling by AMF under drought stress has been observed. Specifically, increased biosynthesis of ABA in AMF mycorrhizal and plants under stressed conditions enhances the establishment of AM symbiosis and improves tolerance to drought.^93^ The ability of AMF to increase ABA biosynthesis under drought conditions helps plants to better manage water stress. ABA is a key hormone in regulating stomatal closure and other drought responses, making this an important adaptation for plant survival in arid environments. The inoculation of AMF with rhizobacteria has been observed to increase the ABA as well as IAA contents in tobacco shoots under drought stress. This suggests a synergistic effect of AMF and beneficial bacteria in enhancing plant stress responses through hormone regulation.^203^ By increasing IAA and cytokinin levels and decreasing ABA levels, AMF promotes the growth of fine roots, which are essential for nutrients and water uptake. This hormonal balance is particularly important after root damage, ensuring rapid recovery and continued growth. In *Lycium barbarum* L. (Goji), the inoculation of AMF has been observed to increase the content of IAA both in leaves and roots. These changes help maintain osmotic balance and improve salt stress resistance. This highlights the impact of AMF in enhancing hormone levels to support plant adaptation to saline environments.^204^

### 5.2. Strigolactone (SL)

**Figure 3.**
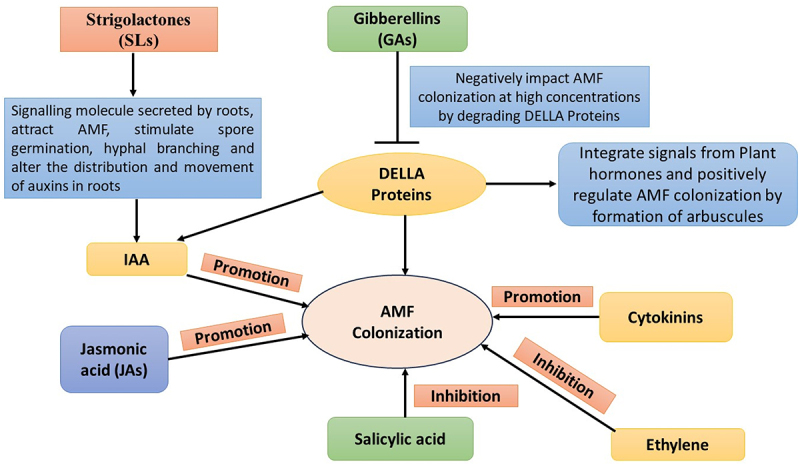
DELLA protein-mediated AMF colonization by plant growth regulators.

Strigolactones (SLs), secreted by plant roots, act as key signaling molecules in the rhizosphere, attracting AMF, stimulating spore germination and hyphal branching, and initiating pre-symbiotic communication.^205^ Strigolactones mainly affect how auxin functions rather than increasing its production. They modify the distribution and movement of auxin, especially in roots, in that way affecting processes such as lateral root growth and root hair formation. Strigolactones can disrupt auxin’s feedback regulation on the positioning of PIN proteins, which influences auxin transport and channeling. Although they do not directly generate auxin, strigolactones are essential in modulating their impact on plant growth and development. Auxins, particularly indole acetic acid (IAA), promote root branching and fungal colonization and are also involved in regulating arbuscule development.^206^ DELLA proteins integrate signals from plant hormones, particularly gibberellins (GAs), which negatively regulate AMF colonization (Figure 3). High GA levels lead to DELLA degradation, reducing AMF colonization, while low GA levels stabilize DELLAs and enhance symbiotic interactions. DELLA proteins, positively regulate the AMF symbiosis by helping in arbuscule formation.^207^ Cytokinins (CKs) contribute to arbuscule development and nutrient exchange, although their role can be either stimulatory or inhibitory depending on the context.^208^ Ethylene generally acts as a negative regulator of AMF colonization, especially at high concentrations, while low levels may support the early stages of interaction.^209^ Jasmonic acid (JA) is implicated in later stages of colonization and may also help induce systemic resistance,^210^ whereas salicylic acid (SA) is often antagonistic, with high levels activating plant defense responses that can suppress colonization.^211^

In both plants with and without AMF colonization, a decrease in the strigolactone (SL) content has been observed under drought stress, suggesting the efficiency of mycorrhizal symbiosis under water-limited conditions.^160^ Auxin response gene *MdIAA24* overexpression in *M*. *pumila* roots demonstrated the enhancement in the mycorrhizal infection by increasing SL content. This leads to an increased rate of mycorrhizal association and higher resistance to drought in transgenic plants.^196^ A negative correlation between low P and SL content has been observed.^212^ *NSP1* and *NSP2*, members of the GRAS transcription factor, have been identified as key mediators of the response to low P by inducing SL biosynthesis genes. These transcription factors also show participation in the AM association signaling pathway in plants.^213^

The carotenoid cleavage dioxygenase (CCD) 7 and 8 gene expression increases under phosphorus-deficient conditions, facilitating the synthesis of strigolactones (SLs) and their secretion by the PDR (pleiotropic drug resistance, efflux pumps) transporter. SLs, in turn, stimulate AMF metabolism and mycelial growth toward the plant roots. Mutations in SL synthesis or transporter genes reduce AMF infection rates, highlighting the importance of these early signals in symbiosis establishment.^214,215^ It has been observed that a mutation in the N-acetylglucosamine transporter (NOPE) 1 prevents AMF from infecting plant roots, which is evident in both *Z. mays* and *O. sativa*. This further suggests that SLs do not alone act as crucial signaling molecules in the pre-association phase.^216^ In *M. sativa*, it has been observed that CCaMK (calcium/calmodulin-dependent protein kinase) and CYCLOPS (cyclops protein) are bound by the DELLA protein, forming a complex activating the GRAS (GAI-RGA-SCR; gibberellic acid insensitive, repressor of ga1–3, scarecrow) transcription factor, RAM1 (Figure 3). The same activation was not observed in the eam1 mutants.^217,218^ A novel kinase, namely arbuscule development kinase, has been identified in *O*. *sativa* (OsADK1), which has been demonstrated to be essential for arbuscule formation.^55^ Certain transcription factors, such as DIP (drought-induced protein) I, NSP (nodulation signaling pathway) 1, 2, and MIG1 (Myc-interacting factor) 1, have also been identified, which play important roles in arbuscular mycorrhizal symbiosis, and any mutations in these transcription factors affect intracellular AMF development. ^219–222^

### 5.3. Gibberellic acid (GA)

Gibberellic acid (GA) biosynthesis is also regulated in plants by P status. GA biosynthesis-related genes are down-regulated by low P stress, while DELLA gene transcription is enhanced.^223^ DELLA proteins repress the GA signaling and have been demonstrated to influence the development of arbuscules. DELLA proteins stunt plant biomass accumulation and development, indicating their role in regulating the extent of mycorrhizal colonization and arbuscule formation with plants.^224^ This suggests that DELLA proteins play a crucial role in modulating plant growth and development in response to AM symbiosis. By repressing GA signaling, DELLA proteins ensure that resources are allocated toward the establishment and maintenance of the symbiotic relationship. The expression levels of GA degradation and signaling genes are substantially altered under the AMF symbiosis, suggesting a complex regulatory mechanism by which GA signaling is modulated to facilitate AM symbiosis.^221^ The downregulation of GA biosynthesis under low P conditions and the concurrent upregulation of DELLA genes suggest a mechanism where plants prioritize symbiotic interactions over, growth under nutrient-stress conditions. This regulatory balance ensures efficient nutrient exchange and symbiotic establishment (Figure 3).

### 5.4. Abscisic acid (ABA)

The modulation of phytohormone concentrations under the arsenic (As) stress was observed under the influence of AMF. This imparts an increased content of IAA along with ABA contents and a decreased content of GA with zeatin riboside. Such regulation helps plants cope with arsenic toxicity by maintaining hormonal balance and reducing stress damage.^225^ The ability of AMF to adjust hormone levels, such as increasing IAA and ABA while decreasing GA and zeatin riboside, helps plants counter the adverse effects of stress due to heavy metals like arsenic (As) toxicity. This underscores the protective role of AMF in contaminated environments.

### 5.5. Polyamines

**Figure 4.**
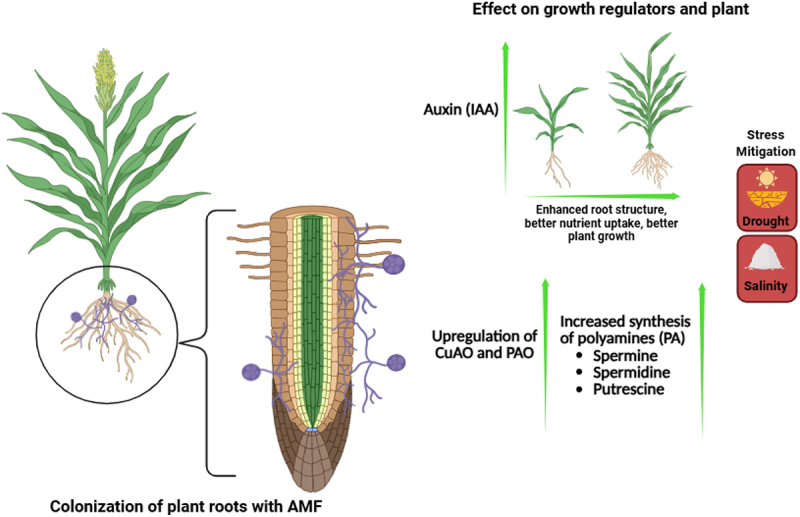
An overview of the involvement of plant growth regulators in plant growth and development and the mitigation of abiotic stress conditions.

Polyamines are organic compounds found in all living organisms, ranging from prokaryotes to eukaryotes. Spermidine (Spd, a tri-amine), spermine (Spm, a tetra-amine), and putrescine (Put, a di-amine) are the three major polyamines that are most prevalent in higher plants. They play a vital role in regulating plant physiology and development, as well as stress management.^226^ During prevailing abiotic stress conditions polyamine have been known to act as osmoprotectants in plants by providing membrane stability, maintaining pH balance, ion homeostasis and reducing chlorophyll loss.^227^ AMF inoculation (*G. margarita*, *F. mosseae*, *R. irregularis*, and *F. constrictum*) in *G. max* under drought stress showed upregulation of spermidine synthase and spermine synthase genes, thereby increasing the polyamine synthesis in the plant. Drought-stressed trifoliate orange (*P. trifoliata*), when inoculated with *F. mosseae* also provides evidence of increased polyamine synthesis by the upregulation of copper-containing diamine oxidase (CuAO), polyamine oxidase (PAO), and putrescine synthases; arginine decarboxylase.^225^ Similar findings were observed when *F. constrictum* was inoculated in *Z. mays* subjected to salinity stress; an increase in polyamine content was observed due to the increased activity of polyamine metabolism enzymes such as spermidine synthase, spermine synthase, and arginine decarboxylase.^133^ Wheat (*T. aestivum*) also mitigates salt stress when subjected to AMF such as *Glomus spp*. by increasing the metabolic activity of SpD and SpS.^154^ Drought resistance in pedunculate oak (*Q. robur*) was observed in the presence of ECM (ectomycorrhizal) fungi by increased synthesis of polyamines (spermidine and spermine), proving the fact that AMF helps plants mitigate stress conditions^95^ (Figure 4).

### 5.6. Nitric oxide (NO)

Nitric oxide is also an important signaling molecule in plants, having a modulatory effect in proline biosynthesis by increasing the activity of Pyrroline-5-carboxylate synthetase (P5CS) and downregulating proline dehydrogenase (ProDH) activity, thereby increasing proline (osmoprotectant) accumulation and helping plants mitigate stress.^228^ Inoculation of *R. irregularis* in *O. sativa* increased nitric oxide synthase (NOS) and nitrite reductase (NR) activity in plants, which in turn helps plants synthesize an adequate amount of proline to help rice plants mitigate salinity stress.^229^ Alleviation of cadmium toxicity in *Z. mays* under the influence of *F. mosseae* was observed, where nitric oxide, having a similar role as that of auxin helps in having elongated root length and modulated membrane transporter activity preventing cadmium to enter the cells proving the detoxification role of NO in heavy metal stress.^230^

## 6. Use of AMF in sustainable agriculture

Leveraging the natural capabilities of soil can minimize the need for chemical inputs like fertilizers and pesticides, which are expensive and environmentally detrimental. Reduction in the usage of chemicals will further lower the cost incurred and decrease the damage to the environment. Lowering the use of water and fuel can cut costs and improve agricultural efficiency. Avoiding ecological contamination safeguards ecosystems, promoting long-term sustainability in agriculture.^231,232^ Implementing as well as monitoring effective management systems ensures the soil remains conducive to crop growth while fostering beneficial soil microbes.

Mycorrhizal fungi, especially AMF, play a vital role in mobilizing nutrients into forms that plants can readily use, which is particularly valuable when nutrient availability is limited. The proliferation of AMF enhances soil quality and structural stability, ultimately promoting better plant growth.^233^ By enhancing nutrient availability, mycorrhizal associations can reduce the need for chemical inputs such as insecticides and fertilizers. Utilizing mycorrhizal fungi efficiently boosts agricultural productivity while minimizing external inputs.^234^ Soil fungi also play a role, such as aiding in decomposition processes and providing essential nutrients to plants.^235^ The symbiosis between plants and AMF is very old and has existed since before plants colonized land. AMF’s coexistence with plants over millions of years suggests a deep-rooted symbiotic relationship that has persisted and evolved alongside terrestrial vegetation. AMF contributes significantly to nutrient cycling and biomass production by soil microbes, enhancing overall soil fertility and plant health.^236,237^ Understanding these relationships and harnessing them are crucial for sustainable agriculture. By optimizing plant genetics and fostering beneficial soil interactions, farmers can improve crop yields while reducing reliance on external inputs and mitigating environmental impacts. Thus, by understanding the historical, environmental and ecological significance of AMF, it can provide valuable insights into their potential and beneficial applications in modern agriculture systems as well as the restoration of ecosystems. Harnessing these beneficial relationships with plants can contribute to sustainable land use practices and enhance soil health for future generations. In a nutshell, integrating mycorrhizal associations into agricultural practices represents a sustainable approach that enhances soil health, reduces environmental impact, and improves crop productivity. These practices are essential for ensuring long-term agricultural sustainability and resilience.

## 7. Conclusion

AMF is a type of mycorrhizal association that penetrates deep into the plant’s root, thereby becoming intricately linked with the physiology of the plant. Being in a symbiotic relationship, it is a win-win situation for both parties, where the plant gets more nutrients and water while the AMF gets its required carbon source. AMF changes root architecture, regulates the transport of water, manages the level of reactive oxygen species through antioxidant machinery, and controls polyamine biosynthesis to impart tolerance against various stresses. Plant hormones strigolactones and auxins are responsible for setting up and maintaining the associations. The current review discusses the significance of AMF in alleviating the trauma of abiotic stress on plants, particularly salinity, dehydration, extreme temperature and heavy metals. The usage of AMF could be of utmost importance in developing sustainable agriculture. AMF contributes to environmental sustainability by improving soil carbon sequestration, reducing erosion, and supporting nutrient cycling. Their potential application in sustainable agriculture presents opportunities to address challenges posed by climate change and unsustainable farming practices, enhancing crop productivity and resilience.
